# Apoptosis and p53 expression in the placental villi of females with unexplained recurrent spontaneous abortion

**DOI:** 10.3892/etm.2013.1399

**Published:** 2013-11-11

**Authors:** DEHUA WEI, QINGHUA WU, HUIRONG SHI

**Affiliations:** 1 Department of Gynecology and Obstetrics, The First Affiliated Hospital of Zhengzhou University, Zhengzhou, Henan 450000, P.R. China; 2 Department of Gynecology and Obstetrics, Puyang People’s Hospital, Puyang, Henan 457000, P.R. China

**Keywords:** p53, unexplained recurrent spontaneous abortion, apoptosis

## Abstract

The aim of this study was to explore the level of apoptosis and p53 expression in the placental villi of patients with unexplained recurrent spontaneous abortion (URSA). Fifty-three pregnant females with URSA and 32 pregnant females who required an induced abortion were selected as the subjects of this study. Placental villus tissues were collected from June 2010 to June 2012 and quantitative polymerase chain reaction (qPCR) and immunohistochemical analysis were performed to determine the mRNA and protein levels of p53 in the placental villus tissues. The level of apoptosis in the tissues was studied using terminal deoxynucleotidyltransferase-mediated dUTP nick end labeling (TUNEL) assay. The mRNA and protein expression levels of p53 in the URSA group were significantly higher than those in the control group (P<0.05). Furthermore, the levels of apoptosis were increased markedly in the URSA group compared with the control group (P<0.05). In conclusion, the placental villi of patients with URSA express a high level of p53, which may result in cell apoptosis and lead to recurrent spontaneous abortion.

## Introduction

Recurrent spontaneous abortion (RSA) is defined as two or more consecutive pregnancy losses prior to 20 gestational weeks (1). Studies have identified numerous causes for RSA, including genetic (2), endocrine (3) and autoimmune (4) causes, which account for ~50% of patients with RSA. The mechanisms for the remaining cases are unexplained and these cases are known as unexplained recurrent spontaneous abortion (URSA). In recent years, a number of studies have shown that a high level of apoptosis in the chorionic villi and decidua is associated with RSA, revealing that apoptosis may be one of the causes of RSA (5,6). p53, a negative cell cycle regulator, is important in numerous biological processes, such as the cell cycle, DNA repair, differentiation and apoptosis (7). p53 has been observed to be expressed abnormally in the chorionic villi and decidua of females with hydropic, spontaneous or missed abortions; however, the expression of p53 in the chorionic villi from patients with URSA has, to the best of our knowledge, yet to be investigated (8–10). In the present study, p53 expression in URSA and the corresponding correlation between p53 expression and URSA were analyzed.

## Subjects and methods

### Subjects

A total of 53 patients with URSA and 32 control volunteers were recruited from the First Affiliated Hospital of Zhengzhou University from June 2010 to June 2012. All patients included in the study exhibited the following clinical characteristics: i) A regular menstrual cycle and menstrual blood volume, with normal color; ii) no chromosomal abnormality or family history of abortion; iii) no reproductive system diseases; iv) negative for cardiolipin, sperm and endometrial antibodies; v) no endocrine diseases; vi) no cardiovascular or venereal diseases; vii) no long-term medication history, no history of radiation therapy and no trauma or drug allergy; viii) no mother-child incompatibility of blood types; ix) no unhealthy habits, such as smoking; and x) no psychiatric history. The age of the 53 patients with URSA ranged between 21 and 40 years (mean, 28.8±7.8 years); the pregnancy duration ranged between 40 and 75 days (mean, 55.3±9.8 days) and the diameter of the gestational sacs ranged between 1.20 and 4.37 cm (mean, 2.65±1.08 cm). The controls were volunteers who came to the hospital for an induced abortion. The age-range of the controls was 20–38 years (mean, 29.1±8.6 years); the pregnancy duration ranged between 39 and 65 days (mean, 53.8±9.4 days) and the diameter of the gestational sacs ranged between 1.17 and 4.55 cm (mean, 2.45±1.11 cm). No significant differences in maternal age, pregnancy duration or gestational sac size were identified between the controls and the patients (P<0.05). This study was conducted in accordance with the Declaration of Helsinki and with approval from the Ethics Committee of the First Affiliated Hospital of Zhengzhou University (Zhengzhou, China). Written informed consent was obtained from all participants.

### Quantitative polymerase chain reaction (qPCR)

Chorionic villus tissues were homogenized in 1 ml TRIzol (Invitrogen Life Technologies, Carlsbad, CA, USA) and 200 μl chloroform was added and mixed. The mixture was subsequently naturally stratified on ice and centrifuged at 15,000 × g for 10 min, prior to the supernatant being transferred and mixed with an equal volume of isopropanol. Following this, the RNA was collected by centrifugation at 15,000 × g for 15 min and washed twice with 75% cooling ethanol, prior to being centrifuged for a final time at 10,000 × g for 10 min. The precipitate was subsequently redissolved in diethylpyrocarbonate (DEPC)-treated sterilized water. The RNA was converted into cDNA using reverse transcription reagents (Takara, Dalian, China) and then used for qPCR.

To examine the endogenous mRNA expression of p53, the qPCR was performed using the following primers: p53-forward: 5′-CCCCTCCTGGCCCCTGTCATCTTC-3′; and p53-reverse: 5′-GCAGCGCCTCACAACCTCCGTCAT-3′. The reaction mixture was prepared with SYBR-Green master mix (Roche Diagnostics, Basel, Switzerland), 500 nmol/l of each primer and 80–100 μg of cDNA, to provide a final volume of 20 μl. qPCR was performed using an ABI Prism 7500 instrument (Applied Biosystems, Foster City, CA, USA) with the following cycle parameters: 30 sec at 95°C, followed by 40 cycles of 3 sec at 95°C and 30 sec at 60°C. The specificity of the product was determined using melting curve analysis according to the manufacturer’s instructions. The data acquired were analyzed using the 2^−ΔΔCT^ method.

### Immunohistochemical analysis

Each tissue sample was paraffin-embedded and cut into 5-μm sections, prior to being mounted on a glass slide and dried for 5 min at 70°C. The slides were deparaffinized in xylene, rehydrated using graded ethanol and washed in phosphate-buffered saline (PBS; 0.2% Tween-20) three times for 5 min each time. PBST was used for all subsequent washes. The tissues were quenched in 3% H_2_O_2_-methanol, washed three times and blocked with PBST containing 10% goat serum for 30 min at 37°C. The slides were subsequently incubated with mouse-anti-human p53 antibody (1:200 dilution; Cell Signaling Technology, Inc., Danvers, MA, USA) at 4°C overnight. Following three further washes to remove excess antibodies, the slides were incubated with diluted goat-anti-mouse peroxidase-conjugated antibody (Santa Cruz Biotechnology, Inc., Santa Cruz, CA, USA) for 30 min at 37°C. The slides were then washed three times, prior to staining with 3,3′-diaminobenzidine (DAB) as the chromogen. Following this, the slides were counterstained with hematoxylin, dehydrated using a graded ethanol series and mounted using mountant. A semi-quantitative method was used to analyze the levels of p53 protein. An average of 10 fields was observed for each specimen at a magnification of ×400. The Motic Med 6.0 Digital Medical Image Analysis System (Motic Instruments Inc., Richmond, Canada) was used for data analysis.

### Terminal deoxynucleotidyltransferase-mediated dUTP nick end labeling (TUNEL) staining

Apoptosis was detected using TUNEL staining, in accordance with the manufacturer’s instructions (TUNEL kit; Roche Diagnostics). In brief, paraffin-embedded sections were deparaffinized and rehydrated as previously described in the immunohistochemistry analysis, prior to being pretreated with proteinase K for 30 min at 37°C and rinsed with PBST three times for 5 min each time. Samples were then incubated with 50 μl TUNEL reaction mixture for 1 h at 37°C in a wet-box. Following a further three washes, 4′,6-diamidino-2-phenylindole (DAPI) was applied for nuclear staining. The sections were subsequently observed under a fluorescence microscope (Olympus BX60; Olympus, Tokyo, Japan). at a magnification of ×400. An average of 10 fields was observed for each specimen. The degree of apoptosis was represented by the percentage of positively stained cells. Slides that were treated in the same manner, although without incubation with the TUNEL reaction mixture, served as negative controls.

### Statistical analysis

The data are presented as the mean ± standard deviation and were analyzed using SPSS 17.0 statistical software (SPSS, Inc., Chicago, IL, USA). Measurement data were compared using a Dunnett’s t-test, while enumeration data were analyzed using a χ^2^ test. P<0.05 was considered to indicate a statistically significant difference.

## Results

### Expression levels of p53 in URSA

**Figure 1:**
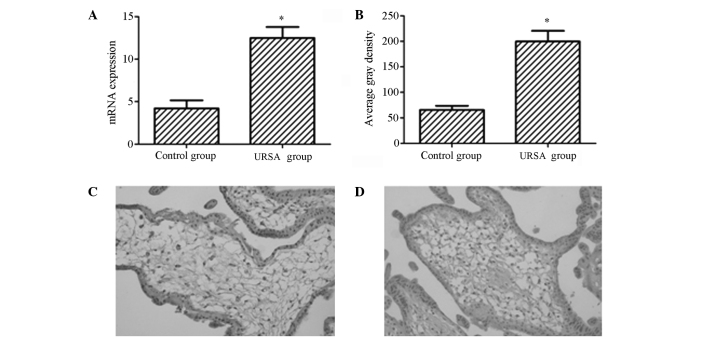
mRNA and protein expression of p53 in the chorionic villus tissues of females with unexplained recurrent spontaneous abortion (URSA). (A) mRNA expression levels and (B) protein expression levels in the control and URSA groups. Immunohistochemical analysis of the (C) control and (D) URSA groups: Staining, 3,3′-diaminobenzidine (DAB); magnification, ×400. ^*^P<0.05 compared with the control group.

In order to validate the variability of p53 expression in females with URSA compared with that in females with a normal pregnancy, qPCR and immunohistochemistry were performed to evaluate the expression levels of p53 in the chorionic villi of the URSA and control groups. The results showed that the mRNA and protein expression levels of p53 were upregulated in the URSA group compared with those in the control group (Fig. 1A and B), with statistically significant differences (P<0.05). As shown in Fig. 1C and D, the p53 protein was observed to be predominantly distributed in the nucleus, appearing as yellow or pale brown particles.

### Apoptotic events in URSA

**Figure 2:**
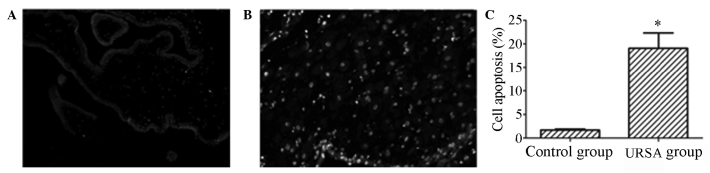
Apoptosis levels of chorionic villus tissues of females with unexplained recurrent spontaneous abortion (URSA). (A and B) Cell nuclei were stained with 4′,6-diamidino-2-phenylindole (DAPI) and apoptosis was revealed using fluorescein isothiocyanate (FITC) staining (magnification, ×400). (A) Control and (B) observation (URSA) groups; (C) Results of the statistical analysis. ^*^P,0.05 compared with the control group.

The level of apoptosis in the chorionic villi of females with URSA was analyzed using a TUNEL assay. As shown in Fig. 2A and B, a significant increase in the number of apoptotic events was observed in the chorionic villus tissues of the URSA group compared with the number in the control group. The results of the statistical analysis are shown in Fig. 2C: The level of apoptosis was demonstrated to be 2.16% in the control group, compared with 19.7% in the URSA group. The difference between the groups was identified to be statistically significant (P<0.05).

## Discussion

RSA is a health problem that affects 1–5% of females of a childbearing age. In ~50% of patients with RSA, the mechanisms remain unexplained. It has increasingly been demonstrated that the occurrence of URSA is associated with a high level of cell apoptosis (6). Apoptosis, also known as programmed cell death, is regulated by a series of genes and is important for cell proliferation and differentiation. It may be that a low level of apoptosis in placental villi and decidual tissues is a normal physiological phenomenon (11) and that RSA occurs when levels of apoptosis are high. A study by Shiraishi *et al*(12) of rat abortion models revealed that the apoptosis level increased significantly in the chorionic villi (placental tissue) of rats with URSA (12), indicating that the RSA may have been due to the high level of apoptosis.

A number of studies have revealed that the abnormal expression of genes involved in apoptosis, such as Fas/Fas ligand (FasL) (13), transforming growth factor (TGF)-β (14), tumor necrosis factor (TNF)-α (15) and Bcl-2/Bcl-2-associated X protein (Bax) (16), in placental villi and decidual tissues is one of the causes of RSA. p53 is an important protein involved in apoptosis and has been shown to participate in cell cycle regulation (17,18). In the present study, the mRNA and protein expression levels of p53 were investigated in the chorionic villus (placental) tissues of females with URSA by qPCR and immunohistochemical analysis. The mRNA and protein expression levels of p53 were observed to be significantly higher in the URSA group compared with those in the healthy control group. This indicated that the RSA may have been due to the abnormal expression of p53 in the chorionic villi. This result was consistent with results from studies investigating other types of abortion (19,20).

To validate the function of p53 in URSA, the levels of apoptosis in the placental villi of patients with URSA were detected using a TUNEL assay. TUNEL is an established method used to detect DNA fragments; DNA fragmentation represents a characteristic hallmark of apoptosis. It was observed that the number of apoptotic events were significantly higher in the chorionic villus tissues of the URSA group compared with the number in the control group, which suggests that the high expression level of p53 resulted in an increased number of apoptotic events, and thus led to RSA.

In conclusion, a high level of p53 expression may result in an elevated level of apoptosis, which may then lead to RSA. However, the detailed regulatory mechanisms require further study.
